# The cultural capitalists: Notes on the ongoing reconfiguration of trafficking culture in Asia

**DOI:** 10.1177/1741659017700947

**Published:** 2017-04-13

**Authors:** Donna Yates, Simon Mackenzie, Emiline Smith

**Affiliations:** University of Glasgow, UK; Victoria University of Wellington, New Zealand; University of Glasgow, UK

**Keywords:** Art crime, cultural heritage, cultural property, illicit antiquities, trafficking

## Abstract

Most analysis of the international flows of the illicit art market has described a global situation in which a postcolonial legacy of acquisition and collection exploits cultural heritage by pulling it westwards towards major international trade nodes in the USA and Europe. As the locus of consumptive global economic power shifts, however, these traditional flows are pulled in other directions: notably for the present commentary, towards and within Asia.

Most analyses of the international flows of the illicit art market have described a global situation in which a postcolonial legacy of acquisition and collection exploits cultural heritage by pulling it westwards towards major international trade nodes in the USA and Europe (e.g. Brodie et al. 2000; Kersel, 2006; Renfrew, 1999). As the locus of consumptive global economic power is shifting, however, these traditional flows are pulled in other directions: notably for the present commentary, towards and within Asia. There have always been ‘internal’ antiquities collecting markets in Asia, largely overlooked by a literature on looting that has been more concerned with westward flows (Byrne, 2016), and some of the dynamics supporting those acquisition practices in Asia are intensifying.

The context for these developments is a practice of illicit trafficking of cultural objects that has emerged as a significant global security concern in recent decades, inspiring a body of interdisciplinary academic research that has embedded this topic within the core of discourses in criminology, sociology, anthropology, cultural studies and law (Brodie, 2006, 2010; Davis and Mackenzie, 2015; Mackenzie, 2011; Mackenzie and Yates, 2016b; Yates, 2014). Of particular concern are antiquities, a subset of cultural objects, which in most cases must be destructively looted from heritage sites, then moving through various smuggling networks and intermediaries and ultimately being sold on the art market (Brodie et al., 2000; Brodie and Tubb, 2002). This looting causes a complete loss of archaeological context and, in turn, the loss of our ability to meaningfully interpret the piece scientifically (Coggins, 1971; Renfrew, 1999). This destructive criminal activity is to the detriment of the primary goal of archaeology: the pursuit of knowledge about the past. Beyond this loss of scientific knowledge, the movement of illicit antiquities on to the art market can be seen as a challenge to political sovereignty and cultural dignity as well as a direct threat to modern religious and social identities (Mackenzie and Yates, 2016b).

## Cultural capitalism: Power, dominance and the flow of Asian antiquities

To simplify what, in trafficking, is often a complex translation of objects through various contexts, antiquities often flow from poor countries to wealthy countries or, perhaps more accurately given the observations concerning internal national collecting mentioned above, from poor communities to wealthy ones. The movement of antiquities from source to market is a process of privatization, usually from some degree of collective custodianship to private ownership. Cases of antiquities looting, trafficking and sale become a simulacrum of global power imbalance and the objects in question can be seen as emotionally charged symbols of post-colonial domination.

The global market for antiquities developed as an extension of Europe’s colonial expansion, embedded in enlightenment ideals of social and cultural capital. The form and function of this market in collecting, investing in and displaying cultural property was developed in the era of colonial occupation of foreign territories to feed European elite demand (Gosden and Knowles, 2001). This demand was first for antiquities symbolic of what were seen as the foundations of European exceptionalism (in particular ‘Classical’ antiquities from Greece, Italy and West Asia), souvenirs of the Grand Tour,^1^ and second for ‘curiosities’ (cultural objects from the Americas, Africa, Oceania and Asia) that fell entirely outside a Western conception, and conceptualization, of culture.^2^

The market allowed elites to convert their economic capital into a form of cultural capital (Bourdieu, 1986) through the long-term project of acquiring cultural objects individually and in batches that were then converted into a collection, which in the symbolic world of high culture represents the collector’s attainment of a particular worldly status (Ortiz, 1994). Thus, the modern international art market, as the inheritor of nearly two centuries of this form of acquisition of symbolic culture through the commodification and recontextualization of cultural objects, is socially and economically structured to benefit the global west/north at the expense of the east/south. It has exploited the cultural heritage resources of poor countries to feed the prerogatives of the world of high culture which has developed predominantly in rich countries.

Exploitative international economic arrangements are hardly unusual. Think, for example, of the low-cost outsourcing of production lines for consumer products, which shifts occupational hazards to low-income communities around the world while increasing profits at the retail market end of the supply chain (Klein, 2000). Comparably, in antiquities trafficking chains, looters in the lower-income countries are exposed to significant occupational risk for scant reward, the value of their illicit labour being appropriated by international traffickers and then most significantly by antiquities dealers and collectors at the top of the market supply chain (Brodie, 1998; Kersel, 2011; Mackenzie and Davis, 2014; Yates, 2015). This market structure involves the layering of cultural power onto capitalist economic power and it is in this way that we refer to those who acquire forms of cultural capital in this way as ‘cultural capitalists’: a term that deconstructs the symbolic work of a crass translation of economic capital into status-through-culture.^3^ In this article, we observe that this pattern is in the process of changing, and we sketch some of the important themes in developing an analytical understanding of this redefinition of forms of power through the lens of geopolitical developments in Asia.

In very basic terms, the argument in our short contribution can be concisely stated. In its postcolonial legacy, the art market continues to be widely perceived as funnelling cultural heritage westwards to major international trading centres in the USA and Europe. However, the primacy of flows in these directions is increasingly becoming challenged by the rise of new economic powers: in the Gulf states and perhaps most notably in China. The narrative of the global trade in cultural heritage as an economic offshoot of colonial programmes of domination and exploitation therefore now demands to be retold. The centres of economic gravity in the world are shifting, and with them the pull of cultural objects towards new loci of wealth and associated status-related symbolic forms of acquisition of cultural heritage collections; an acceleration of local antiquities collecting processes which have been observed as subsisting at a lower level in Asia for some time (Byrne, 2016).

To fill out this shifting model of trade flows in cultural heritage, and the looting and trafficking that are the criminal effects of the long shadow cast by a demand for this kind of cultural consumption, will require detailed regional analyses to chart the new relationships being developed between countries. In Asia, this will involve a fine-grained understanding of the development of economic and cultural values in China and how these can explain its internal and external (i.e. import) markets in cultural property collecting: too intricate for the present sketch. It will also require looking again at trade centres that have been noted to be ‘transit portals’ for illicit antiquities, like Singapore and Hong Kong (Polk, 2000), the stories of which have until recently been told as part of the imperialist and then postcolonial narrative we have mentioned: essentially as facilitation points in the flow of objects ex-Asia to the West (Murphy, 1995). This view now needs to be recast.

This article is based on the cumulative work of the Trafficking Culture research project, including observation and interview research in the art market in Hong Kong and China, and similar ‘market-end’ interview research with dealers, collectors and other market specialists outside Asia who have in recent years experienced the effects of the financial drivers of the international market on their capacities to buy from, and sell to, sources and destinations in Asia. More information about the research programme, its methodological approaches, data and publications, is available at: www.traffickingculture.org.

## Neoliberal colonialism: Asian objects in the West

The presence of Asian cultural objects on the historic art market can be understood as part of the history of the globalization of world trade that developed out of the mentalities of colonialism. For example, China’s longstanding and diverse artistic tradition created minor artworks specifically for the Western market which had little appeal on the Chinese market, but were crafted to satisfy 18th and 19th century American and European demand (see, for example, Mudge, 1981). This Western ‘export’ style was largely decorative, and cultural objects originating in South, South-East and East Asia continue to be strongly associated with decoration in Western art markets. Even today, Asian objects tend not to be classified as ‘antiquities’ on the global art market, no matter their age: this term is reserved for the Classical and Biblical civilizations that the West has adopted as its own lineage. Instead, dealers, auction houses and even museum exhibits still separate ‘Oriental art’ or now ‘Asian art’, from ‘ancient art’, within which standard terminology we can read legacies of racism similar to those which are easier to spot such as in the application of the still widely-used labels of ‘primitive art’ or ‘tribal art’ to cultural objects sourced from Africa, the Americas and Oceania.^4^

By the mid-19th century and certainly into the 20th century, a growing Western interest in the history of Asian culture, as well as a shift in Western art market taste, placed increasing amounts of value on ancient authenticity. Here, the antiquities from throughout Asia from a time before Western contact and subsequent domination were objects used in the service of a conceptualization of ancient Asian civilizations as somehow separate from the modern Asian civilizations that the West encountered: these past cultures were constructed as more advanced and more respectable than those of the people who were presently being dominated (see Meyer and Brysac, 2015 for this era of Western collecting in China). Contemporary inhabitants were thus seen as improper custodians of their own past and were characterized as not caring for their ancient cultural patrimony (Cuno, 2014). Movement of these objects to Europe and the USA, even via the market and outside the concept of the museum, was portrayed as saving the objects (Cuno, 2008; Fitz Gibbon, 2005).

By contrast, at the same time foreign collectors were busy saving cultural objects from the purportedly undeserving locals, local collectors in Asia were finding justification, and in some cases government complicity, for their acquisitive cultural practices in the idea that they were saving objects from ‘avaricious foreign collectors’ (Byrne, 2016)! The growing discourse around national cultural heritage, a process critically termed ‘heritization’ by those who see some degree of artifice and opportunism in its claims (Panella, 2014), has been implicated in expanding the allure of artefacts for local collectors, as opposed to increasing the likelihood of protection of archaeologically relevant cultural objects in the ground (Byrne, 2016).

Dealers engage in market-making activities. As fresh flows of looted cultural heritage objects were opening up from Asia, Western collectors had to be untethered from their attachment to the classical antiquities with which they were more familiar and whose ‘Classical’ Western forms they appreciated. Dealers worked on their clients to educate them into seeing the beauty of the statues that were being smuggled out of Asia from places like Nepal, Thailand, Cambodia and India. In the combination of a developing appreciation of the aesthetic, and the developing fixation with authenticity in preference to fakes, impressions and innuendoes, Asian art found its international market. This process, which dealers tell as an educational interaction and work of benevolent edification of their client collectors (Mackenzie, 2005), can of course quite properly be seen as the systematic pressing of economic priorities into the practices of art appreciation being learned by a cohort of ready consumers for the symbolic goods, the value of which is written through this fundamentally capitalist ideological exercise.

In a recent paper on wildlife crime, an international black market which is sometimes compared to antiquities trafficking (Mackenzie and Yates, 2016a), the authors have proposed a conceptualization of ‘neoliberal colonialism’ which they see as a process of commodification (‘converting everything into alienable property’) and commercialization (Peterson et al., 2016). For cultural property markets, commodification (cf. Kopytoff, 1986) and commercialization of the sometimes ineffable inscribed meanings and values of the artefacts in question are clearly identifiable processes. However, the version of commercialization observable in the creation and working of these markets is the selling of social meaning along with the chattels themselves (Žižek, 2009). As with other status goods, what is being bought and sold is both earthly material and the metaphysics of social power. Traders in diamonds, luxury cars, high-end cigars and so on profit from the same tricks of symbolic enhancement of worldly goods (Naylor, 2011). For antiquities the cultural meaning is more complex than for many of those other status purchases, being imbued with the possibility that, as the famous collector George Ortiz put it, one might in buying the objects also ‘acquire the spirit behind them’ (Ortiz, 1994). It is this relation between cultural symbolism, economic power and competitive international relations that makes the changing global flows of illicit antiquities such an interesting subject of study.

Understandably, there has been significant criticism, both within Asia and abroad, of the removal of Asian antiquities ‘for their own protection’ by cultural capitalists who have argued that the objects were not being valued or protected within their original context. This assertion assumes that Western definitions of both value and preservation in relation to cultural objects are universal, thereby denying differing interactions with heritage if they do not conform to Western norms. Such an argument is a form of intellectual colonialism. That the commodification of such objects may go against local tradition and legislation was and is viewed by the traffickers and by the market as of little concern, both because the so-called protection of antiquities was seen as a motivating factor that was above and beyond the law in its calling (Mackenzie and Yates, 2016a), and because those engaging with the looting and smuggling of these antiquities could reasonably assume that they would never be punished for their actions.

This lingering postcolonial view of the art market was embraced by the contemporary art market, but panned by the critical movements of the 1960s and 1970s (Coggins, 1969, 1972). That critique can be seen particularly with respect to the embedding of cultural property within ideals of national sovereignty via such international bodies as the United Nations, and the growing concept of a heritage of all humankind which, at least on some level, constructs private ownership of the past as a violation of universal access to shared heritage (Mackenzie and Yates, 2016b). Yet the adoption of the 1970 UNESCO *Convention on the Means of Prohibiting and Preventing the Illicit Import, Export and Transfer of Ownership of Cultural Property* did little to arrest the flow of Southeast and East Asian antiquities out of Asia and into the West. Conflict and instability in the region, particularly in Southeast Asia, created a fragile security situation which was exploited by market participants and facilitators (Mackenzie and Davis, 2014). Governmental de-legitimization of certain periods of historical and cultural manifestations in China and Cambodia led to further heritage loss (Davis and Mackenzie, 2015). Ongoing poverty, funding shortfalls, corruption and ineffectual governmental protection programs, when manifested in the context of a continuous market demand for antiquities, continue to threaten the safety and security of cultural objects in much of Asia to this day.

## Inter-Asian trafficking of illicit antiquities

The biggest market for illegally excavated relics is now shifting to China. (Nasim Javed, owner of the Zaitun Art Gallery, Peshwar, Pakistan, quoted in Khan, 2016)

In light of the general narrative of cultural property theft and loss outlined above, many countries in Asia not unreasonably place blame on Western demand for the continued theft of their antiquities. Yet there is an indication that now the East-to-West, South-to-North model of the flow of illicit cultural property is shifting, aligning more with regional networks and more contemporary formulations of power and control. The economic dominance of China within Asia and beyond appears to be having a parallel effect on the accumulation of cultural capital, with China becoming a site of consolidation for much of Asia’s cultural heritage wealth. This can be conceptualized as, to some extent, an importation of Western values in relation to conceptions of the symbolic aspects of cultural capital, with affluent Chinese collectors buying up art and antiquities from weaker states falling within the Chinese sphere of influence.

When an antiquity is looted in Nepal, India or Cambodia, the West will likely still be blamed by locals and activist observers who often describe London, Paris or New York as the ultimate destination for the stolen piece. However, China is emerging as a strong and at times more likely source of art market demand (Harris, 2015). A growing number of antiquities looters and traffickers arrested regionally within Asia, and even globally, cite China as the ultimate destination of the stolen cultural objects they aimed to sell into the international market, e.g. the Lord Raghunath idol theft in Himachal Pradesh by a Nepali intending to smuggle the piece to China (*Business Standard*, 2016); a seizure of 2000 China-bound ancient coins in Pakistan in 2014 (Shahid, 2014); and a shipment of illicit sandlewood, artefacts and idols stopped in Chennai Port that was meant to travel to China via Dubai (Kumar, 2016).

If China, like Europe and the USA before it, is pulling international flows of cultural property towards its orbit, does this suggest a striving for cultural property market domination and thus the aim of a form of cultural custodianship of all of Asia, mapping on to its economic domination of the region? And does the process of growing this collecting market itself represent a straightforward translation of the European model of the art market into a specific Asian context, or is it the development of an independent, entirely non-Western art market with different ideals, goals and ethical outcomes? Questions like these seem pertinent points of departure for future research.

## Looking to the future: Cultural capitalism, Asia style

Our tentative answers here would be that in the process of China’s synchronization with the world (Ren, 2010) it has engaged with neoliberalism and globalization on its own terms. Ideas of culture and status that have been constructed around the Western interpretation of the global art market have not been wholly rejected, rather they have translated, as China has begun to achieve domination both as a buying power and in terms of its legal and diplomatic repatriation demands for cultural objects held in collections overseas. With a renewed appreciation for its past, China’s internal art market is centred around the accumulation of the attributes of class through culture, and it appears to be in the process of changing a market in looted antiquities characterized by a strong international component into a regional inter-Asia trade flow in that continent.

China’s consistent claims for repatriation of cultural objects abroad have pushed for recognition of China’s status on the cultural property playing field, using the repatriation claims not only as an assertion of national sovereignty, but also as an instrument for promoting cultural identity and national unity (Liu, 2016). One of the best-known repatriation claims concerns the bronze zodiac sculptures of the Old Summer Palace, looted during the Second Opium War by British and French troops. Five of these Imperial zodiac sculptures were bought abroad by Chinese state-owned museums or by Chinese private buyers who subsequently donated the objects to Chinese museums. Two of these sculptures, the rat and rabbit, were auctioned by international auction house Christie’s in 2009. Despite China’s condemnation, Christie’s proceeded with the auction. The winning bidder identified himself as an advisor of China’s National Treasures Fund and refused to pay for the objects in protest, stating it was his patriotic duty to stop the sale. The owner of Christie’s subsequently purchased the two zodiac figures from the consignor for an undisclosed amount and repatriated the objects in 2013. China had tightened import and export regulation for Christie’s as retribution for its role in what was widely regarded as the sanctioning of colonial looting and historical injustice. However, after repatriating the zodiac figures, Christie’s was allowed its inaugural auction in Shanghai that same year. The returned zodiac figures became symbols of a rising China overcoming Western imperialism (Kraus, 2004). The political and economic game China plays by participating in the global art market becomes clearer in such examples.

So China synchronizes its national cultural property market and accompanying values and tastes with the global cultural property trade on its own terms. Shifting tastes, developed alongside observation of Western-constructed standards of cultural capital, have ensured that Chinese billionaire collectors such as Liu Yiqian and Wang Jianlin are dominating the art market locally as well as globally. They are stimulated by a nationalistic ideology of repatriation and historical reverence, as well as a desire for recognition of Chinese identity and culture. Cultural property is one among the instruments available to reposition nationalist pride and authority.

Over the last decade, China has become the epicentre of the global cultural property trade, and will likely continue to extend its power and influence in this area. The asymmetry in the exchange of cultural objects between source and market countries is being rectified in the case of China, as the poles of the market adjust to reconfigurations of economic power. As a result, the cultural property trade will be forced to revise its Eurocentric premises, adapting to more appropriate local conceptions, traditions and values in respect of new economic powers. These historical and observational notes on the reconfiguration of trafficking culture in Asia provide a starting point for researchers in understanding the nature of contemporary trends.

## Notes

1. A mid-17th to mid-19th century rite of passage for young European males which brought them to the cultural centres of ancient civilizations that were conceived of as being the progenitors of European culture: the Greeks, the Romans, the Biblical cultures. Thompson (1963), among others, sees the Grand Tour as a practice in maintenance of cultural hegemony by the ruling class, which we believe can be seen in the removal of antiquities from these regions as both a process of acquiring symbolic culture and of cultural domination.

2. Epitomized by the creation of ‘Cabinets of Curiosities’ by elite collectors, containing strange specimens of nature and culture which fell outside of the normal European conception of civilized self. They symbolized the collector’s social status as both an aesthete and a controller of the known universe (see, for example, Impey and MacGregor, 2001; Mauriès, 2011).

3. The term ‘cultural capitalism’ has been used in a comparable way, different in its inflections to the thrust of the present article, to account for the broader theme of contemporary consumer capitalism commodifying and selling cultural experiences. This involves layering certain cultural and moral meanings into the basic commodities sold, as part of a neoliberal process that subsumes culture within economy, using the former in service of the latter (Žižek, 2009).

4. For example, at the time of writing, Sotheby’s Auction House maintains a department called ‘Ancient Sculpture and Works of Art’, which features only objects from the Classical and so-called Biblical civilizations, with a separate department and separate sales for ‘Indian, Himalayan & South East Asian Art’ containing pieces that are just as ancient. Christie’s Auction House has a department for ‘Ancient Art and Antiquities’ (Christie’s Auction House, n.d.), which only features ‘works of art from across the ancient Mediterranean world’, and separate departments for ‘Chinese Ceramics & Works of Art’ as well as ‘Indian, Himalayan & Southeast Asian Art’ which feature objects that can easily be termed ‘ancient’ mixed with more recent works.
